# IgA vasculitis mimicking drug-induced skin reaction and infectious colitis in an elderly patient: A case report

**DOI:** 10.1097/MD.0000000000027726

**Published:** 2021-11-05

**Authors:** Sung Kyun Yim, Seung Young Seo

**Affiliations:** Department of Internal Medicine, Research Institute of Clinical Medicine of Jeonbuk National University-Biomedical Research Institute of Jeonbuk National University Hospital, Jeonju, Republic of Korea.

**Keywords:** elderly, Henoch–Schönlein purpura, IgA vasculitis

## Abstract

**Rationale:**

Immunoglobulin A vasculitis (IgAV) in adults is rare and shows worse symptoms and prognosis. In real-life clinics, IgAV in elderly patients may be difficult to diagnose because of its rarity and other common diseases to consider. This study reports a case of IgAV mimicking drug-induced skin reaction in an elderly patient.

**Patient concerns:**

A 74-year-old female without any underlying disease presented to our emergency department as she was suffering from lower abdominal pain and diarrhea for 3 weeks. The patient was diagnosed with infectious colitis, and antibiotic treatment was administered at a local clinic. At presentation, the patient had rashes on both lower legs that developed after the antibiotic treatment, which was assumed as a drug eruption. Although antibiotic treatment was continued, the patient had persistent abdominal pain, diarrhea, hematochezia, and rashes. Proteinuria was developed on urinalysis.

**Diagnoses:**

Infectious colitis, IgAV.

**Interventions:**

Sigmoidoscopy revealed easily bleeding erythematous mucosal lesions from the descending colon to the rectum. IgAV was suspected, and thus skin biopsy was performed. Histological findings of the skin biopsy showed leukocytoclastic vasculitis, which is compatible with IgAV. A nonsteroidal anti-inflammatory drug was administered for abdominal pain. The patient showed persistent proteinuria and a systemic steroid (prednisolone 50 mg [1 mg/kg]) was started.

**Outcomes:**

After administration of the nonsteroidal anti-inflammatory drug, the patient's abdominal pain was resolved rapidly. Under systemic steroid treatment, the patient showed significant symptomatic improvements, and after 2 weeks, the skin and colonic mucosal lesions were completely resolved.

**Lessons:**

We present a case of adult-onset IgAV, which was initially diagnosed with infectious colitis and drug eruption. The history of skin reaction development after antibiotic treatment and the rarity of IgAV in elderly patients masked the diagnosis of IgAV. Despite its rarity, IgAV should be highly suspected in elderly patients with rashes, proteinuria, and signs of colitis.

## 1 Introduction

Immunoglobulin A vasculitis (IgAV), formerly known as Henoch–Schönlein purpura, is a common systemic vasculitis that is usually diagnosed in children and has a self-limited course in most cases.^[1,2]^ Common clinical presentations are palpable purpura, arthralgia, abdominal pain, and kidney disease, which is a classical tetrad, with other manifestations such as gastrointestinal bleeding and pulmonary hemorrhage.^[1–4]^ In contrast to IgAV in children, adult-onset IgAV is rare and shows worse symptoms and prognosis primarily due to renal involvement.^[2,5]^ The underlying cause of IgAV remains unclear, but triggering factors, such as infection, drugs (eg, antibiotics), and vaccination were reported.^[1,6]^

In real-life clinics, because of its rarity and other common diseases to consider, IgAV in elderly patients presenting with abdominal pain or rashes may be difficult to diagnose, especially with a preceding infectious disease. Herein, we present a case of an elderly patient with IgAV, which was initially diagnosed as infectious colitis and drug-induced skin reaction after antibiotic treatment. This case report was approved by the Institutional Review Board of Jeonbuk National University Hospital (IRB No. 2021–05–025), and informed consent was obtained from the patient for publication.

## 2 Case report

**Figure 1:**
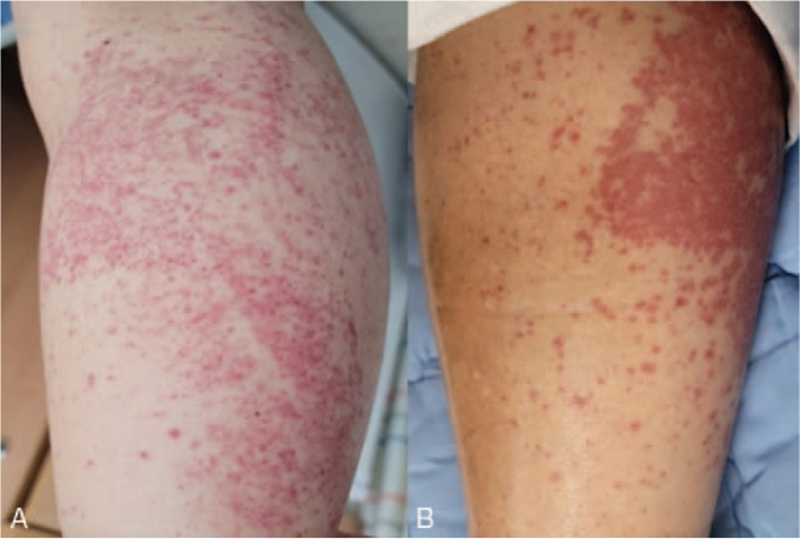
Skin lesions of the patient upon presentation in the emergency department (A) and after admission to the ward (B). Diffused erythematous rashes were present in both lower legs.

**Figure 2:**
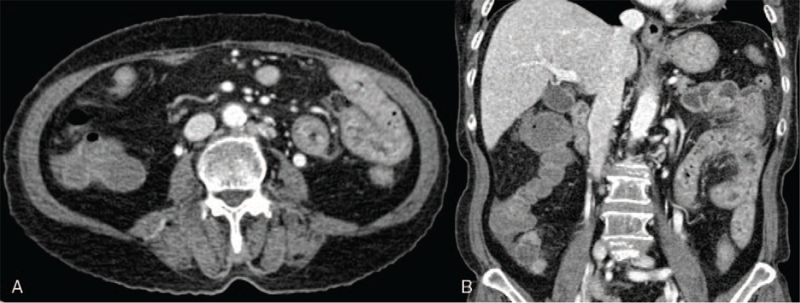
Abdominal computed tomography at presentation reveals edematous wall thickening of the small bowel and loose stools in the colon.

**Figure 3:**
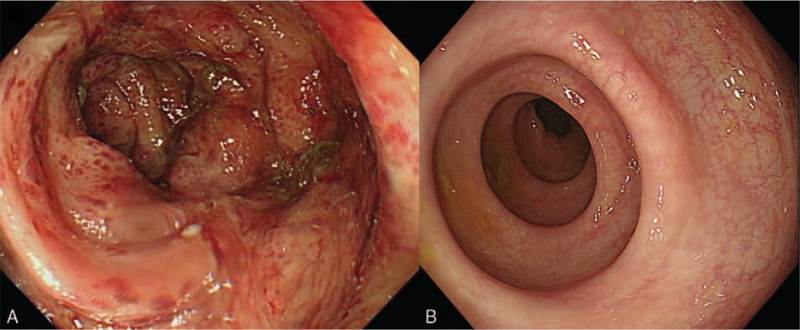
Sigmoidoscopic findings on day 5 showed multiple patchy erythematous mucosal lesions and erosions from the rectum to the distal descending colon (A). Follow-up sigmoidoscopy 2 weeks after steroid treatment showed complete healing of the mucosal lesions (B).

A 74-year-old female without any underlying disease visited the emergency department as she was suffering from lower abdominal pain and diarrhea for 3 weeks. The patient was hospitalized at a local clinic and was treated with intravenous antibiotics (third-generation cephalosporin), but showed no improvement. Upon presentation, the patient was febrile (37.5°C), but other vital signs were stable. The patient had diffuse erythematous rashes on both lower legs, which occurred after antibiotic treatment and were assumed to be a drug allergy (Fig. 1). The Dermatology department was consulted, and because drug-induced skin reactions could not be ruled out, conservative management was recommended. Laboratory examination revealed the following: leukocytes, 22,460 cells/μL (neutrophils, 84.0%); hemoglobin, 11.0 g/dL; platelets, 440 × 10^3^/μL; aspartate aminotransferase, 19 IU/L; alanine aminotransferase, 11 IU/L; blood urea nitrogen, 6 mg/dL; creatinine, 0.50 mg/dL; lactate dehydrogenase, 358 IU/L; and C-reactive protein, 66.75 mg/L. Stool leukocyte was positive, but no organism was isolated in stool culture. Abdominal computed tomography did not show definite inflammation of the colon, but an increased amount of loose stools and air-fluid levels suggested colitis (Fig. 2). Focal segmental wall thickening of the small bowel was also observed. Based on clinical symptoms and signs such as abdominal pain, diarrhea, fever, leukocytosis, positive stool leukocytes, and abdominal computed tomography findings, infectious colitis was suspected. Intravenous antibiotics (piperacillin plus tazobactam) were administered. However, the patient showed ongoing abdominal pain, and hematochezia occurred. For further diagnostic evaluation, sigmoidoscopy was performed. Multiple patchy erythematous mucosal lesions and erosions that easily bleed were observed in the rectum and the distal descending colon (Fig. 3A). Moreover, proteinuria, which was not present at the time of presentation, was detected on urinalysis. Because the patient had purpura, proteinuria, and abdominal pain with mucosal lesions on sigmoidoscopy, IgAV was suspected. A skin biopsy was performed for diagnosis. Histological findings showed leukocytoclastic vasculitis, which is compatible with IgAV. Under the diagnosis of IgAV, a nonsteroidal anti-inflammatory drug was administered for abdominal pain, and the patient's abdominal pain was resolved rapidly. However, the patient showed persistent proteinuria, and under the consultation of the Nephrology department, a systemic steroid (oral prednisolone 50 mg [1 mg/kg]) was started. Then, the patient showed significant symptomatic improvements. After 2 weeks, the skin and colonic mucosal lesions were completely resolved (Fig. 3B). Afterward, the patient was discharged and was followed up as an outpatient for 6 months. Oral prednisolone (50 mg, 1 mg/kg) was continued for 1 month and was tapered over 4 months. An angiotensin-converting enzyme inhibitor (ramipril 2.5 mg) was also administrated. During the follow-up, proteinuria was gradually improved and eventually resolved at the fourth month of follow-up.

## 3 Discussion

IgAV is a type of immune-mediated vasculitis wherein IgA plays a central role.^[7,8]^ The diagnosis of IgAV is based on its clinical manifestations, such as palpable purpura, arthralgia, abdominal pain, and kidney disease.^[9]^ However, in those with atypical presentation and in adults, biopsies for the affected organ are required. Although the precise cause of IgAV is unclear, several triggering factors, such as infections, drugs, chemicals, and vaccinations, were reported.^[1,6,7]^ In children, IgAV is known to be frequently preceded by an infection, such as viral and bacterial infections. However, in adults, drugs and chemicals, such as antibiotics, nonsteroidal anti-inflammatory drugs, and angiotensin-converting enzyme inhibitors, are more common triggering factors.^[7]^ The incidence of IgAV varies according to age and the affected population. In children, the annual incidence of IgAV varies from 13.50 to 21.75 per 100,000.^[7,9,10]^ However, IgAV is relatively rare in adults with an annual incidence of 0.12 to 1.40 per 100,000.^[7,9]^ Our patient had an episode of abdominal pain with diarrhea before presenting to our institution and was diagnosed with infectious colitis. After treatment with antibiotics, skin lesions developed, which were assumed to be an allergic reaction toward the antibiotics. Whether the infection or the antibiotic was the triggering factor for this patient is unclear, but the patient's history masked the diagnosis of IgAV. As in this case, the diagnosis of IgAV in adults can also be overlooked due to its rarity in real-life clinics.

In adult patients with IgAV, renal involvement is more common (50%–85%), and the chance of progression to end-stage renal disease is higher, which indicates that the long-term prognosis of IgAV is worse in adults than in children.^[1,3,7,9]^ Due to its rarity, data on IgAV in the elderly are limited. However, some studies have reported that renal involvement is more severe and shows a poorer prognosis in elderly patients (≥60 years) than in younger patients (19–60 years).^[11,12]^

The incidence of gastrointestinal involvement varies among studies.^[1,3]^ In a Korean study involving 160 patients with IgAV, adult patients have shown a higher rate of gastrointestinal symptoms (56.3% in children vs 62.5% in adults).^[3]^ Common gastrointestinal symptoms are abdominal pain, nausea, vomiting, melena, or hematochezia. Intussusception may occur in severe cases.^[1,2,13]^ Reported endoscopic findings of IgAV are edema, erythema, petechial, erosion, and ulceration.^[9,13]^ In our patient, although IV antibiotics were maintained, abdominal pain persisted and hematochezia developed. Endoscopy showed similar lesions to the reported findings of gastrointestinal manifestation of IgAV that resolved after systemic steroid treatment. Thus, the gastrointestinal findings can be assumed as a complication of IgAV. Although endoscopic findings of IgAV are not disease-specific, as in our patient, adult patients showing findings of colitis in endoscopy with accompanied proteinuria and/or skin lesions should be suspected for IgAV.

IgAV often presents with a self-limited course and treatment usually focuses on symptom control and supportive measures.^[2,8]^ Common treatments for IgAV include resting, adequate hydration, and pain control using acetaminophen or nonsteroidal anti-inflammatory drugs. However, as previously mentioned, adult-onset IgAV has a more severe course and may need further treatment. Systemic steroids with or without immunomodulatory drugs (eg, colchicine, dapsone, mycophenolate mofetil, cyclophosphamide, rituximab) are used in life-threatening cases, but the efficacy of treatment is still controversial, especially in older patients, due to lack of randomized control trials.^[2,8]^ Also, precise indication alongside optimal dose and duration of systemic steroid is not well established in adult IgAV. The evidence supporting the use of steroids for IgAV is in part based on trials performed on patients with IgA nephropathy.^[2]^ Because of the same reason, there is no specific guideline for adult IgAV. A recent European guideline for children and younger adults recommends a systemic steroid treatment in case of orchitis, cerebral vasculitis, pulmonary hemorrhage, severe abdominal pain, hematochezia, and other life-threatening involvements.^[14]^ Oral prednisolone/prednisone of 1 to 2 mg/kg/day or pulsed intravenous methylprednisolone (10–30 mg/kg with a maximum of 1 g/day on three consecutive days) is recommended in severe cases. Other treatment modalities may include angiotensin-converting enzyme inhibitors for renal involvement, renal transplantation for end-stage renal disease, and surgery in case of severe gastrointestinal bleeding or perforation.^[8]^ In our case, the patient showed hematochezia and proteinuria. A systemic steroid (oral prednisolone 50 mg, 1 mg/kg) and angiotensin-converting enzyme inhibitor (ramipril 2.5 mg) were administered and the patient recovered.

## 4 Conclusion

We present a case of adult-onset IgAV, which was initially diagnosed with infectious colitis and drug eruption. The history of skin reaction development after antibiotic treatment and the rarity of IgAV in elderly patients masked the diagnosis of IgAV. Despite its rarity, IgAV should be highly suspected in elderly patients with rashes, proteinuria, and signs of colitis.

## Author contributions

**Conceptualization:** Seung Young Seo.

**Data curation:** Sung Kyun Yim, Seung Young Seo.

**Resources:** Sung Kyun Yim, Seung Young Seo.

**Validation:** Seung Young Seo.

**Visualization:** Sung Kyun Yim, Seung Young Seo.

**Writing – original draft:** Sung Kyun Yim.

**Writing – review & editing:** Sung Kyun Yim, Seung Young Seo.
